# Small bowel edema and lymphocytic duodenitis as severe reversible gastrointestinal toxicity of selpercatinib in *RET* fusion–positive non–small cell lung cancer: a case report

**DOI:** 10.3389/fonc.2023.1201599

**Published:** 2023-07-10

**Authors:** Daniela Scattolin, Elena Scagliori, Antonio Scapinello, Alberto Fantin, Valentina Guarneri, Giulia Pasello

**Affiliations:** ^1^ Medical Oncology 2, Veneto Institute of Oncology (IOV), IRCCS, Padova, Italy; ^2^ Department of Surgery, Oncology and Gastroenterology, University of Padova, Padova, Italy; ^3^ Radiology Unit, Veneto Institute of Oncology (IOV), IRCCS, Padova, Italy; ^4^ Pathology Unit, Veneto Institute of Oncology (IOV), IRCCS, Padova, Italy; ^5^ Gastroenterology Unit, Veneto Institute of Oncology (IOV), IRCCS, Padova, Italy

**Keywords:** NSCLC, RET, selpercatinib, gastrointestinal toxicity, small bowel

## Abstract

**Introduction:**

Rearranged during transfection (*RET*) gene rearrangements occur in 1%–2% of non–small cell lung cancer (NSCLC). Because of the results of the study LIBRETTO-001, selpercatinib has been approved as the first-line treatment for patients with *RET* fusion–positive advanced NSCLC. Selpercatinib demonstrated to be well tolerated. Despite this, gastrointestinal adverse events (AEs) are frequently reported, and no clinical-radiological and endoscopic features and their impact in terms of treatment discontinuations, interruptions, and dose reductions have been described so far.

**Case report:**

A 37-year-old never-smoker woman was treated in our institution with selpercatinib for a *RET* fusion–positive NSCLC. After 9 months of treatment, the patient referred abdominal pain of grade (G) 2, associated with nausea of G2, bilious vomiting of G3, and weight loss of G1. At computed tomography scan, the presence of important bowel wall thickening, free ascitic fluid, mesenteric congestion, and stranding was detected. The patient underwent an anterograde enteroscopy extended to jejunum with detection of lymphocytic duodenitis with sub-mucosal edema. Selpercatinib treatment was temporary interrupted with complete resolution of the symptoms and then re-administered with dose reduction, without relapsed of the gastrointestinal toxicity after 120 days.

**Conclusion:**

To our knowledge, this is the first case report of a patient with NSCLC treated with selpercatinib outside a clinical study who developed severe gastrointestinal toxicity characterized by small bowel edema and lymphocytic duodenitis, leading to treatment interruption and dose reduction. The gastrointestinal AE has been described by a radiological, endoscopic, and histopathological point of view. Further investigations are needed to better identify pathological mechanisms of gastrointestinal toxicity for an appropriate AE management.

## Introduction

The presence of alterations of the rearranged during transfection (*RET*) gene has demonstrated an emerging role in the treatment scenario of oncogene-addicted non–small cell lung cancer (NSCLC) and thyroid cancer in the last years. *RET* gene rearrangements occur in 1%–2% of NSCLC and in 5%–10% of patients with diagnosis of papillary thyroid cancer (1), whereas *RET* mutations occur in more than 95% of hereditary and approximately 50% of sporadic medullary thyroid cancer (2). Patients with *RET*-positive advanced NSCLC (aNSCLC) were initially targeted with *RET* multi-kinase inhibitors (MKIs), frequently leading to off-target adverse events (AEs) and drug dose reductions or discontinuations (2). Thus, in the last years, the potent and highly selective *RET* inhibitors selpercatinib and pralsetinib have been developed for the treatment of patients with *RET* fusion–positive aNSCLC or thyroid cancer (3–8). In particular, selpercatinib is a novel, ATP-competitive, and highly selective small-molecule inhibitor of *RET* kinase with activity against *RET* fusions and activating point mutations and the ability to cross the central nervous system with antitumor activity in the brain (3), including leptomeningeal metastases (9). Because of the results of the LIBRETTO-001 study (3, 4), selpercatinib has been approved by the European Medicines Agency for patients with *RET* fusion–positive aNSCLC not previously treated with *RET* inhibitors and as a second-line treatment for patients affected by *RET*-altered thyroid cancer. Among patients with treatment-naïve aNSCLC, selpercatinib showed an objective response rate (ORR) of 84% (95% CI, 73 to 92) and a median progression-free survival (mPFS) of 22.0 months; in platinum-based chemotherapy pretreated patients, the ORR was 61% (95% CI, 55 to 67) and the mPFS was 24.9 months (10). Because of these results, *RET* fusions are in the Olympus of those driver alterations that should be analyzed in aNSCLC (9). However, the Italian regulatory agency Agenzia Italiana del Farmaco (AIFA) restricted the indication of selpercatinib to patients with *RET* fusion–positive aNSCLC previously treated with platinum-based chemotherapy.

The most common grade (G) ≥ 3 AEs reported with selpercatinib were hypertension (19.7%), alanine aminotransferase increased (11.4%), aspartate aminotransferase increased (8.8%), diarrhea (5.0%) and electrocardiogram corrected QT (QTc) interval prolonged (4.8%) (10). Although gastrointestinal AEs are frequently reported (4, 10, 11) in patients treated with selpercatinib, the clinical-radiological and endoscopic features of gastrointestinal toxicities are not always well described, such as their impact in terms of treatment discontinuations, interruptions, and dose reductions (9).

We report what it seems the first case of a patient treated with selpercatinib for a *RET* fusion–positive aNSCLC who developed severe gastrointestinal toxicity characterized by small bowel edema and lymphocytic duodenitis, leading to treatment interruption and dose reduction.

## Case report

**Figure 1:**
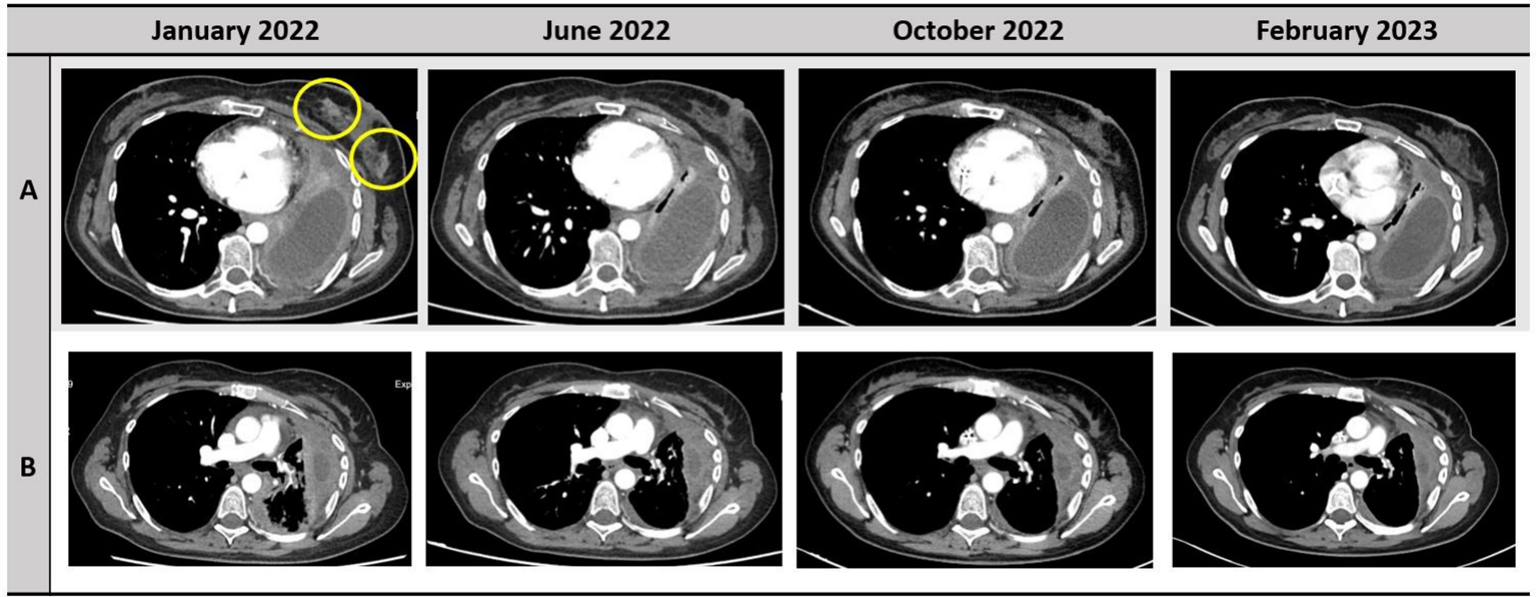
Disease response to treatment with selpercatinib. The responses of left breast nodules and pleura are depicted in the row A and B, respectively.

**Figure 2:**
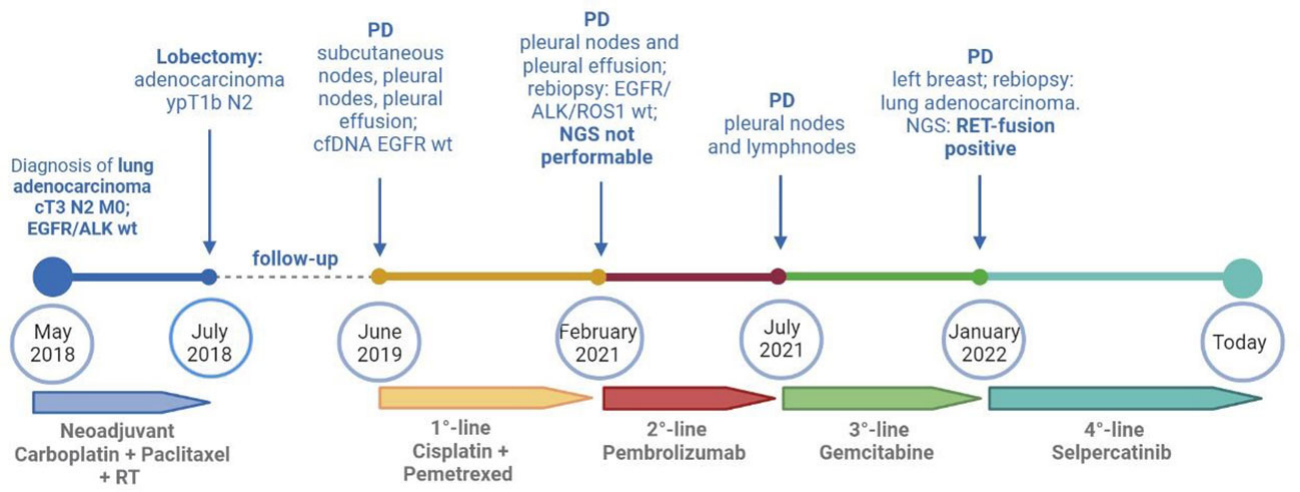
Timeline of treatments received by the patients from diagnosis to today. cfDNA, circulating free DNA; NGS, next-generation sequencing; PD, progressive disease; RT, radiotherapy; wt, wild type. Created with BioRender.com.

A 37-year-old never-smoker woman was referred to our Unit in May 2018 after the diagnosis of a stage IIIB (cT3 cN2 according to Tumor-node-metastasis (TNM), VIII edition) adenocarcinoma of the left lung. The patient had no comorbidities, her performance status (PS) was 0 according to Eastern Cooperative Oncology Group (ECOG), and she had no family history of cancer. After our institutional multidisciplinary discussion, the patient underwent neoadjuvant chemotherapy with carboplatin area under the curve (AUC) 2 and paclitaxel at 50 mg/sm with concurrent radiotherapy of the left lung (64 Gy in 32 fractions) and subsequent lobectomy of the left upper lobe, with final diagnosis of adenocarcinoma ypT1b N2, *EGFR* and *ALK* wild type, and Programmed death-ligand 1 (PD-L1) (tumor proportion score) of 60%. In June 2019, during follow-up, multiple nodules of the thoracic wall, pleural nodes, and pleural effusion were detected; a liquid biopsy was performed, excluding the presence of *EGFR* mutations. Afterward, the patient started a treatment with cisplatin at 75 mg/sm and pemetrexed at 500 mg/sm for four cycles, followed by pemetrexed maintenance, with partial response of disease. In February 2021, a volumetric increase of the pleural nodes and effusion was detected at the radiological examination. Thus, the patient underwent thoracoscopy with rebiopsy of the pleural nodes, confirming the presence of adenocarcinoma metastasis; no alterations of *EGFR*, *ALK*, or *ROS1* were detected. Next-generation sequencing (NGS) extended to other molecular target was not performed because of insufficient material. From April to July 2021, the patient was treated with pembrolizumab at 200 mg, achieving a disease progression in lymph nodes and pleural nodes. Subsequently, the patient underwent a third-line chemotherapy with gemcitabine at 1000 mg/sm. During the treatment, the patient referred the appearance of palpatory nodules of the left breast; she underwent a biopsy of the breast nodules in December 2021 with confirmation of lung adenocarcinoma metastasis. Because of the progressive disease, a FoundationOne panel of NGS analysis of the tissue was performed, and a *RET* fusion (*KIF5B-RET*) was detected. The patient was asymptomatic and in good clinical conditions (PS ECOG 0). In January 2022, a treatment with selpercatinib at 160 mg bis in die (BID) was started. Selpercatinib was initially administered in class C not negotiated, until its approval by the AIFA for national health system reimbursement. The patient underwent partial response of disease at the first radiological assessment (**Figure 1**). Detailed timeline of treatments administered is depicted in **Figure 2**.

**Figure 3:**
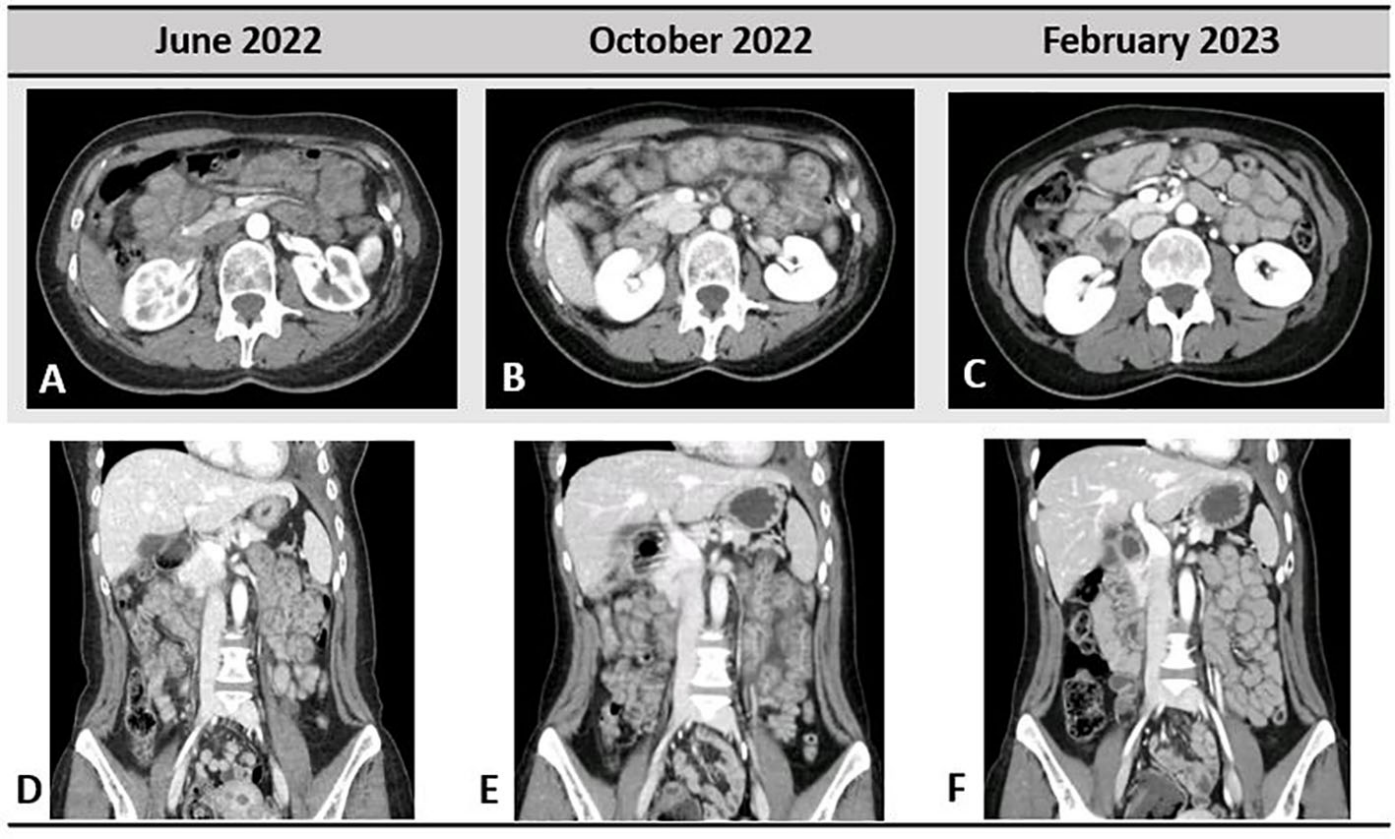
Gastrointestinal toxicity to selpercatinib; very light small bowel thickening was present in June 2022, when the patient started to refer abdominal pain **(A, D)**. In October 2022 **(B, E)**, important bowel wall thickening, mesenteric congestion, and stranding are shown. Complete resolution of the radiological pathological findings in February 2023 is depicted in **(C, F)**.

**Figure 4:**
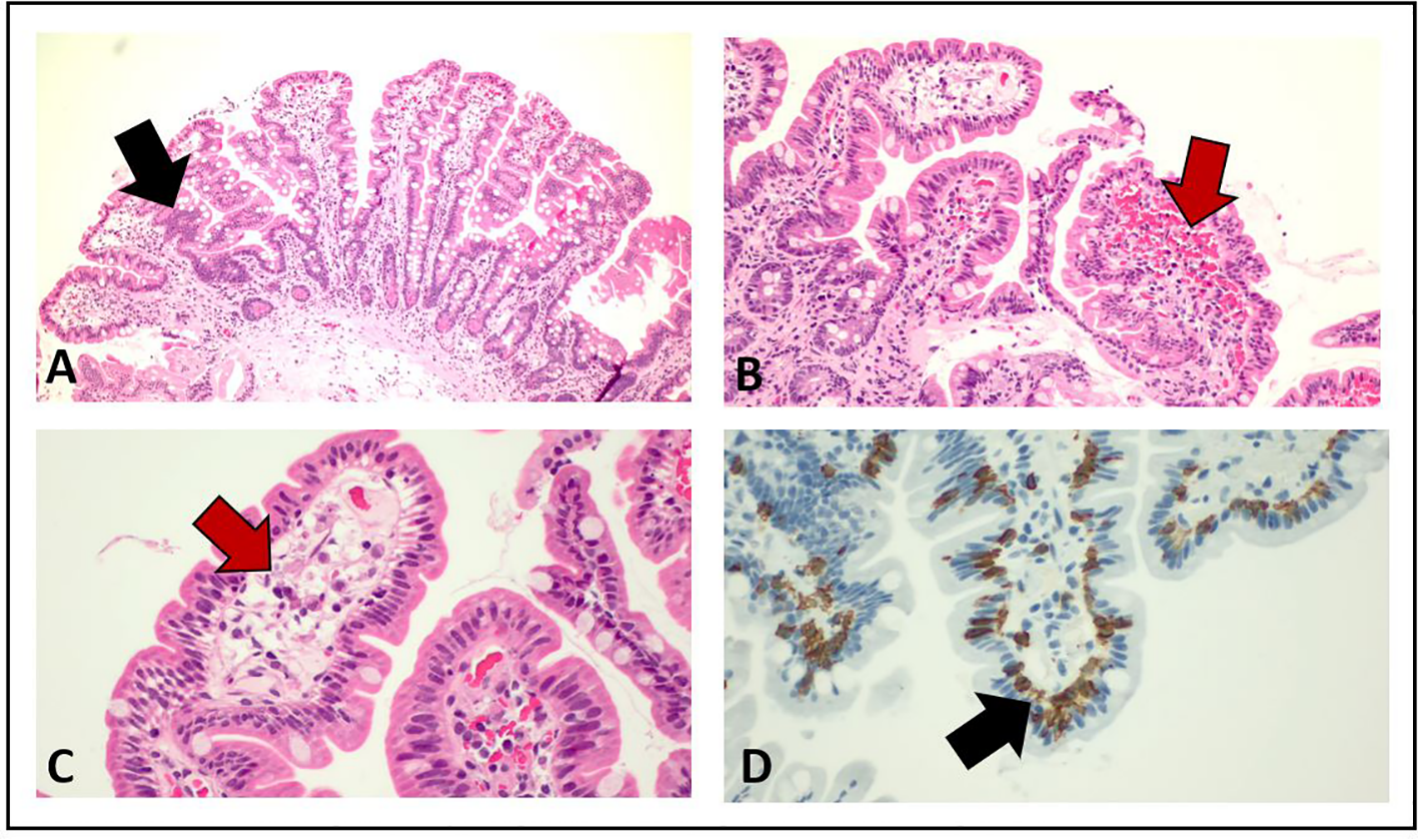
Pathological examination of duodenum and jejunum biopsies from esophago-gastro-duodenoscopy. The red arrows in **(B)** and **(C)** show the presence of submucosal edema, whereas the black arrows in **(A)** and **(D)** indicate the increased number of intraepithelial lymphocytes T CD3+ and CD8+.

Starting from June 2022, the patient referred abdominal pain of G1 with worsening to G2 in September 2022, associated with nausea of G2, bilious vomiting of up to G3, and weight loss of G1. At physical examination, no abnormal abdominal findings were observed. Because of the weight loss with a resulting weight of less than 50 kg, selpercatinib dose was adapted to 120 mg BID. The subsequent computed tomography (CT) scan documented the presence of important bowel wall thickening, free ascitic fluid, mesenteric congestion, and stranding (**Figure 3**). No increased in number or size of mesenteric lymph nodes was detected. To better understand the gastrointestinal symptoms of the patient and to exclude the presence of disease progression in small bowel, an anterograde enteroscopy extended to jejunum with multiple biopsies was performed. At the final histology report, a type 1 (according to Marsh modified classification) lymphocytic duodenitis with sub-mucosal edema was diagnosed; in particular, an increased number of intraepithelial lymphocytes T CD3+ and CD8+ were found in the mucosa of duodenum and jejunum (**Figure 4**).

Suspecting gastrointestinal toxicity due to selpercatinib, the treatment was temporary interrupted. The patient underwent complete resolution of bilious vomiting and nausea after 2 days and abdominal pain after 15 days; 3 days later, selpercatinib was re-administered at first level reduction dose of 80 mg BID. No recurrence of gastrointestinal AEs was referred by the patient after 120 days from the resume of treatment.

At the CT scan performed after 4 months, the patient had complete resolution of the bowel wall thickening, free ascitic fluid, mesenteric congestion, and stranding previously observed (**Figure 3**).

This case has been reported to the Italian pharmacovigilance system with the code RNF 909925.

## Discussion

In the last decades, the development of new drugs targeting specific molecular alterations has changed the history and management of patients with oncogene-addicted NSCLC. Among all the genetic alterations detectable in NSCLC, *RET* fusions are present in a small cohort of patients, around 1%–2% (1). *RET* is a proto-oncogene encoding a transmembrane glycoprotein receptor tyrosine kinase normally activated by the interaction with a soluble neurotrophic factor [glial cell line–derived neurotrophic factor (*GDNF*), neurturin (*NRTN*), or artemin (*ARTN*)] and a co-receptor of the *GDNF* family receptor–α (*GFRα*). The complex formed by *GDNF*–*NRTN*–*ARTN* and *GFRα* binds *RET* extracellular domain determining heterodimerization and auto-phosphorylation of intracellular tyrosine kinase domains. This interaction leads to the activation of signaling pathways normally implied in cell proliferation, growth, differentiation, and survival. These pathways include *RAS*–*MAPK*, *PI3K*–*AKT*, *PKC*, and *JAK*–*STAT*. *RET* rearrangements are caused by the fusion between the C-terminal region of *RET* and the N-terminal region of other genes as *KIF5B*, *CCDC6*, and *NCOA4*, resulting in a chimeric fusion protein constitutively active, with consequent aberrant downstream signaling of cell proliferation (12). Because of its interaction with the *GDNF*, *RET* has an important role in the development and function of the enteric nervous and renal system (13).

Selpercatinib is currently used for the treatment of patients with *RET* fusion–positive NSCLC or *RET*-altered thyroid cancer, after the positive results of the clinical trials LIBRETTO-001 (3, 4, 8, 10). In the last updated analysis of LIBRETTO-001, selpercatinib was confirmed to be well tolerated by patients with NSCLC: 8% discontinued treatment because of AEs, with 3% considered by the investigator to be related to selpercatinib (10). The safety of selpercatinib was confirmed also in the real-world study SIREN, in which G3 ≥ 3 AEs occurred in 24% of patients, including increased liver enzyme levels (10%), prolonged QTc time (4%), abdominal pain (4%), hypertension (4%), and fatigue/asthenia (4%). No patient discontinued selpercatinib treatment for safety reasons, 40% of patients required dose reduction, and 26% temporary interrupted the treatment, but no patients permanently discontinued selpercatinib due to Treatment-related AEs (TRAEs) (11). Despite gastrointestinal toxicity has been largely documented in clinical trials and real-world setting (3, 4, 8, 11), no radiological, endoscopic, or histopathological detailed characteristics have been described so far. Our patient was radiologically assessed at the occurrence of the worse grade of gastrointestinal toxicity, and the CT scan showed the presence of small bowel thickening, free ascitic fluid, mesenteric congestion, and stranding. Similar radiological findings have been reported by Tsang et al. (14) in a small cohort of patients treated with selpercatinib for thyroid cancers from LIBRETTO-001. Among the 20 patients enrolled in this retrospective analysis, 10 (50%) experienced gastrointestinal toxicities during selpercatinib with a median time of 41 days (range, 15–91 days) from treatment start. At the CT scan, the patients with gastrointestinal toxicity had common features allowing the authors to define a score. The radiological criteria of the score include presence of peritoneal free fluid, bowel wall thickening, mesenteric congestion, stranding defined as the increased density of mesenteric fat, and increased number, size, or morphologically abnormal mesenteric lymph nodes. At baseline, no difference in radiological scores between later symptomatic or asymptomatic patients was observed. At symptoms onset, a score of 1 or more was described in seven of the 10 symptomatic patients and in four of the 10 asymptomatic ones. Similarly, our patient had a radiological score of 1 with a very light small bowel thickening and concurrent G1 abdominal pain, whereas a score of 4 was observed 4 months later when the abdominal pain became more severe with associated G2 nausea, G3 bilious vomiting, and G1 weight loss. Furthermore, the pathological findings observed in small bowel biopsies of our patient are in line with those reported in three of the 20 patients retrospectively observed by Tsang et al.; in particular, mucosal edema with vacuolated aspects was a common histology report, whereas intraepithelial lymphocytic infiltration was not underlined in the previous report. The radiological score developed by Tsang et al. has some limitations because of its lack of validation and its low specificity. Indeed, the radiological features of the score are similar to a large spectrum of gastrointestinal injuries from different causes. Furthermore, the score has been developed in patients with thyroid cancers and not enlarged to patients with NSCLC so far. Despite this, the presence of peritoneal free fluid, bowel wall thickening, mesenteric congestion, stranding, and increased number, size, or morphologically abnormal mesenteric lymph nodes should warn the clinician about a potential gastrointestinal toxicity of selpercatinib. Drug interruptions or dose reductions should be then taken in consideration to recover the symptoms and, subsequently, to allow a treatment resumption, thus avoiding permanent discontinuation. A validated score in patients with NSCLC has not been developed so far and should be useful to better stratify the gastrointestinal AEs during selpercatinib and to help clinician in differential diagnosis of radiological pathological abdominal report.

Nowadays, the pathological mechanism of gastrointestinal toxicity due to selpercatinib has not been clearly explained. Despite this, a hypothesis could be done considering the role of *RET* expression for development, maturation, and maintenance of the autonomic and enteric nervous systems, as mentioned before. *RET* expression is abundant in the intestinal epithelium during embryonal development and remained only in a subset of enteroendocrine and enterochromaffin cells in adult enteric tissues. Thus, *RET* signaling is very important in adults for gastrointestinal tract motility and secretion (15). As a consequence, loss-of-function mutations of *RET* can cause bowel dysfunctions. An example of this condition could be seen in the hereditary Hirschsprung’s disease, characterized by aganglionosis of the gastrointestinal tract (16, 17). It is tempting to speculate that the inhibiting role of selpercatinib could result in a gastrointestinal disfunction with a mechanism similar to that of Hirschsprung’s disease.

However, gastrointestinal toxicity has been reported as constipation (22%) and diarrhea (12%) in patients treated with pralsetinib, a *RET*-inhibitor with a mechanism similar to selpercatinib, but no cases of abdominal pain or nausea and vomiting were reported in the ARROW trial (6). Furthermore, a retrospective multicenter study showed chylous effusions in 7% (15 of 217) of patients treated with selpercatinib, at lower frequencies in patients treated with other MKIs, but no chylous effusions were observed in patients treated with pralsetinib (18). Moreover, when properly explored at the CT scans, chylous effusion has been retrospectively observed in a high proportion (80%) of patients with medullary thyroid cancer treated with selpercatinib, as depicted by Prete et al. (19) Thus, further investigations are needed also to explore the reason of this different gastrointestinal involvement with pralsetinib and other MKIs and to clarify whether this type of gastrointestinal toxicity is due to a class-wide or to a tyrosine kinase inhibitor (TKI)-specific effect.

In conclusion, to our knowledge, this is the first case report describing small bowel edema and lymphocytic duodenitis in a patient with NSCLC treated with selpercatinib. In our case, the gastrointestinal AE to selpercatinib has been described by a radiological, endoscopic, and histopathological point of view. Future investigations are needed to better understand the pathological mechanisms of gastrointestinal toxicity for an appropriate AE management also preventing drug interruptions or dose reductions. Furthermore, the development of a validated clinical-radiological score extended to patients with NSCLC could be useful for clinicians in the differential diagnosis process.

## Patient’s perspective and informed consent

The emergence of the gastrointestinal disturbances had a high impact on the quality of life of the patient who demonstrated a high compliance in undergoing further investigation for the differential diagnosis of the symptoms. The patient understood the importance of the treatment with selpercatinib and accepted the management purposed with temporary interruption first and dose reduction later. After the resumption of symptoms and selpercatinib re-administration, her quality of life improved.

The patient of this case report provided consent for the anonymous publication of the report.

## Data availability statement

The raw data supporting the conclusions of this article will be made available by the authors, without undue reservation.

## Ethics statement

The studies involving human participants were reviewed and approved by Ethical Committee of Veneto Institute of Oncology IRCCS Padova. The patients/participants provided their written informed consent to participate in this study. Written informed consent was obtained from the individual(s) for the publication of any potentially identifiable images or data included in this article.

## Author contributions

DS: conceptualization, planning, data collection, figure design, paper writing ES: data collection, figure design, paper approval AS: data collection, figure design, paper approval AF: data collection, figure design, paper approval VG: paper supervision and final approval GP: conceptualization, planning, paper writing, paper supervision and final approval.
